# Energy evolution law and catastrophic failure prediction of Luohe formation sandstone during rupture process experienced one freeze-thaw cycle

**DOI:** 10.1371/journal.pone.0321656

**Published:** 2025-05-08

**Authors:** Liang Zhang, Jianxi Ren, Mengchen Yun, Yichen Zhang, Bolong Liu, Kun Zhang

**Affiliations:** 1 School of Architecture and Civil Engineering, Xi’an University of Science and Technology, Xi’an, China; 2 School of Civil Engineering, Shaoxing University, Shaoxing, Zhejiang, China; Shenyang Jianzhu University, CHINA

## Abstract

To explore the energy damage evolution law and catastrophic failure characteristics of sandstone in Luohe Formation after thawing, the uniaxial compression tests on sandstone after one freeze-thaw cycle with different freezing temperatures were conducted. The mechanical properties and failure mode of thawed sandstone were analyzed. Furthermore, the energy evolution and its sensitivity to freezing temperature during deformation and failure were investigated. Subsequently, a model was proposed to predict the catastrophic failure time of the thawed sandstone in the Luohe Formation. The results showed that the peak strength and elastic modulus of thawed sandstone decrease with the decrease of freezing temperature. The failure modes of the thawed sandstone can be classified into axial splitting failure, shear failure, and tensile-shear mixed failure. Additionally, with the decrease of freezing temperature, the strain energy release rate and dissipation energy growth rate of rock samples decrease. Energy damage process of the Luohe Formation sandstone after thawing is divided into stages of minor damage, deceleration damage, damage steadily increase, damage accelerated increase, and damage sharply increase. The thawed sandstone catastrophic failure presents a clear critical power-law singularity behavior, and its critical singularity characteristic value *β* is roughly 0.7±0.1. The proposed catastrophic failure prediction method yields a prediction time of imminent rock failure (*t*_*f*_^*p*^=4.356 min) that is very close to the actual failure time (*t*_*f*_=4.376 min) at freezing temperature of -10 °C, and the prediction results are good and stable.

**Highlights**

(1) The degree of local rock block peeling and surface particle shedding of thawed sandstone becomes more and more serious with the decrease of freezing temperature.
(2) The energy damage evolution process of thawed sandstone is divided into stages of minor damage, deceleration damage, damage steadily increase, damage accelerated increase and damage sharply increase.
(3) The range of critical singularity characteristic value β of thawed Luohe Formation sandstone is found to be roughly 0.7±0.1.
(4) A catastrophic failure time prediction mode for the thawed sandstone in Luohe Formation was presented according to the critical power-law singularity characteristics.

**List of symbols**

E_*t*_ Total energy

E_e_ Elastic strain energy

E_d_ Dissipated energy

E_*0*_ Initial elastic modulus

*E*_*u*_ Elastic modulus of rock unloading

*σ*_*1*_ First principal stress

*σ*_*2*_ Second principal stress

*σ*_*3*_ Third principal stress

*ε*_*1*_ First principal strain

*ε*_*2*_ Second principal strain

*ε*_*3*_ Third principal strain

$\varepsilon_{1}^{e}$ Elastic parts of first principal strain

$\varepsilon_{2}^{e}$ Elastic parts of second principal strain

$\varepsilon_{3}^{e}$ Elastic parts of third principal strain

*σ*_*1i*_, *σ*_*1i-1*_ Stress values of any two adjacent points in the stress-strain curve

*ε*_*1i*_, *ε*_*1i-1*_ Strain values of any two adjacent points in the stress-strain curve

n Total number of sampling points on the stress-strain curve

*E*_*dc*_ Critical energy dissipation value

*β* Critical power-law singularity index

*Ω* Response variable

*k* Proportionality constant

*λ* Control variable

*λ*_*f*_ Control variable of rock failure

t Evolution time of the control variable

t_*f*_ Failure time

D Energy damage variable

t_*f*_ Failure time

D Energy damage variable

$\dot{D}$ E nergy damage variable rate

$\ddot{D}$ Energy damage variable acceleration

## 1 Introduction

**Fig. 1:**
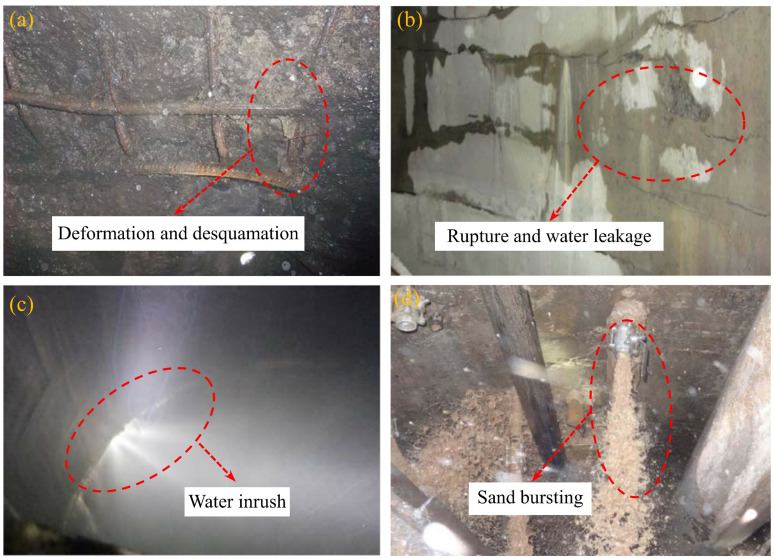
Mine disasters of frozen vertical shaft after thawing: **(a)** Concrete protective layer’ deformation and desquamation; **(b)** Local Rupture and water leakage of shaft lining; **(c)** Water inrush in frozen shaft; and **(b)** Sand bursting of freezing shaft wall.

The western coal mine region, as an important coal production base in our country, mainly crosses the Mesozoic Cretaceous and Jurassic strata in the mine construction. Among them, the Cretaceous strata are mostly composed of sandstone in Luohe Formation, characterized by weak cementation, large pores, distinct bedding, numerous fractures, low strength and susceptibility to weathering, bringing remarkable challenges to the infrastructure construction of coal mine in the western region [1,2]. Artificial freezing method was widely used in the construction of vertical shaft projects in western coal mines due to its unique advantages in ensuring the safety of coal mine shaft construction [3,4]. However, the rock strata of Luohe Formation will undergo deformation under the action of in-situ stress after thawing the artificial frozen vertical shaft, resulting in coal mine disaster such as deformation and rupture, local water leakage, water inrush and even shaft flooding (Fig. 1), which seriously threaten the production safety in western mines. Therefore, it is essential to study the mechanical properties, failure modes, energy evolution and catastrophic failure characteristics of the Luohe Formation sandstone after thawing to ensure the safety and stability of the frozen vertical shafts of large-tonnage coal mine in western area.

The extent of soft-hard and low-temperature has a significant effect on the damage-fracture mechanism of rocks [5,6,7,8]. The effects of low-temperature on mechanical properties and deformation fracture mechanism of freeze-thaw rocks and frozen rocks have been investigated through laboratory tests [9,10,11]. For example, Lu et al. [12] pointed out that the strength difference between rock samples with different structural plane angles tended to decrease with the cell pressure and freeze-thaw cycles. Liu et al. [13] investigated the mechanical and permeability properties of the sandstone induced by freeze-thaw, and found that the initial permeability increased with the freeze-thaw cycles. Liu et al. [14] established a damage evolution model of the frozen sandstone to reflect the temperature effect by using a power function to fit the cumulative AE count. Zhou et al. [15] studied damage features of sandstone subjected to freeze-thaw cycles and revealed the influence of freeze-thaw cycles on the peak stress, microcracks and AE activity. These studies have drawn some beneficial conclusions on the effect of freeze-thaw cycles and low-temperature, but the effect of freezing temperature on the damage and failure characteristics of sandstone of weak cementation in Luohe Formation subjected to one freeze-thaw cycle is still unclear.

In addition, energy accumulation and energy release occur evidently during the full process of rock deformation and failure, and energy dissipation theory has been widely used to study the energy conversion law of rock in loading and unloading processes. It was found that the release of accumulated strain energy plays an important role on the deformation and failure process of rock [16,17]. Meanwhile, the loading mode [18], stress path [19,20], loading and unloading rates [21], fissure inclination and lengths [22,23], confining pressure [24,25], seepage pressures [26] and water content [27] also significantly affect the energy evolution of rocks. Furthermore, several studies on the energy evolution of the rocks under freeze-thaw cycles based on the energy theory were investigated. Zhang et al. [28] investigated the effects of freeze-thaw cycles on the development of strain energy, and demonstrated that the total strain energy tended to decrease after freeze-thaw cycles. Wang et al. [29] found that the rate of energy reduction at different stress thresholds increased after 60 cycles of freeze-thaw. Wang et al. [30] investigated energy-driven damage and fracture evolution of granite specimens induced by freeze-thaw and proposed a coupling damage evolution model base on freezing-thaw and mechanical damage. Li et al. [31] revealed the influence of freeze-thaw and cyclic loading on energy evolution of red sandstone, and energy storage and dissipation of rock under coupling effect show a linear laws. These studies have provided valuable insights into the energy-driven damage and fracture evolution during rock deformation and failure, however, the energy evolution features and its temperature effects of weakly cemented Luohe Formation sandstone subjected to one freeze-thaw cycle under static stress are still far from fully understood.

The suddenness of rock instability and failure often lead to serious engineering accidents, and it shows obvious precursory characteristics can be used as an effective means to predict rock deformation and failure [32]. For the precursory characteristics of imminent rock failure, many scholars have investigated the infrared temperature [33], acoustic emission (AE) signals [34], fracture process zone (FPZ) [35] and the critical slowing down characteristics [36] of rock failure by using infrared imaging and acoustic emission technology. For example, Khan et al. [37] predicted early failure point (EFP) of sandstone with different water contents based on infrared radiation and complex energy evolution during loading. Subsequently, Cheng et al. [38] proposed model can reproduce and predict time-dependent failure behaviors (such as the failure mode, strength, and failure time). Zhou et al. [39] examined the precursory features of rocks failure under the action of freeze-thaw by applying the Critical Slowing Down (CSD) theory, and noted that the AE signals had a CSD phenomenon. In addition, the response variable change rate (deformation, damage, etc.) relative to the control variable tends to infinity and showing obvious characteristics of critical power-law singular accelerated development when the rock approaches the catastrophic failure point [40]. Hao et al. [41] studied the power-law singular characteristics of the rock failure, and proposed a method for predicting the failure time of material catastrophic failure. Liang et al. [42] studied the damage and failure of ceramic coating materials and proposed a damage and catastrophic failure model on the ceramic coatings. Zhou et al. [43] investigated the physical controls on the scatter of exponents in the critical power law relation, and found that the critical power law exponents range from -0.5 to -1.0. Xie et al. [44] proposed a model for predicting rock failure time based on Bi-LSTM recurrent neural network model combined with Attention mechanism. Till now, there are relatively few historical studies investigating the failure precursory characteristics of weakly cemented soft rocks. Especially, the critical power-law singular characteristics of response quantity of the sandstone in Luohe Formation after thawing has not been reported widely, and the prediction model of catastrophic failure time for thawed sandstone has not been established.

Therefore, this study focuses on sandstone in Luohe Formation of the artificial freezing vertical shaft of the western mining areas, and a series of fast freeze-thaw test with different freezing temperatures are conducted using the multi-function freeze-thaw testing machine. Subsequently, the uniaxial compression test of the thawed sandstone are carried out to study the mechanical properties, deformation characteristics and failure modes. By analysing the energy evolution characteristics of the Luohe Formation sandstone under different freezing temperatures, the energy conversion mechanism and the freezing temperatures effect of thawed sandstone during deformation and failure are revealed. Besides, the energy damage evolution mechanism and critical power-law singularity characteristics of thawed sandstone under uniaxial compression is discussed. A prediction model is then established to predict the catastrophic failure time of the thawed sandstone in the Luohe Formation, and the effectiveness and the rationality are verified using the experimental data. The results of this study not only provides in-depth insights into rock damage and fracture induced by one freeze-thaw cycle under uniaxial compressive loading but also offers an important theoretical basis and technical support for the disaster prevention after thawing of frozen vertical shaft in Luohe Formation sandstone.

## 2 Experimental principle

### 2.1 Rock sample preparation

**Fig. 2:**
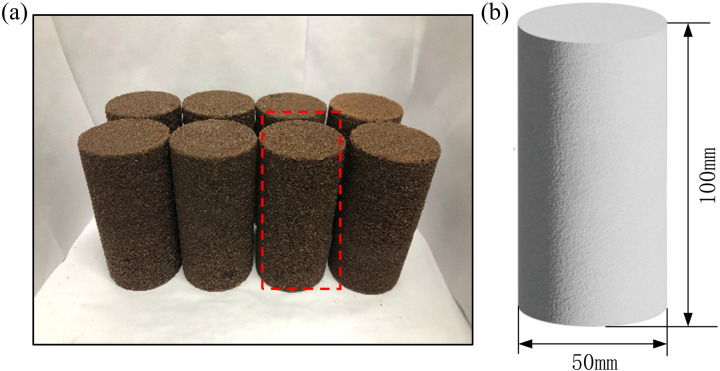
Experiment samples: **(a)** Some of sandstone in Luohe Formation; and **(b)** Specimen dimensions.

The Luohe Formation sandstone used in the test were collected from the construction site of freezing vertical shaft in west mining areas, which were processed into a standard cylinder cylinders with a diameter of 50 mm and height of 100 mm. The parallelism deviation at both ends of the rock sample is less than 0.05mm, and the axis deviation is less than 0.25°. The natural density, water absorption rate, and longitudinal wave velocity of the sandstone in Luohe Formation range is 1.85~1.99 g·cm^3^, 7.19%~10.68% and 1.22~1.85 km·s^-1^, respectively. It can be seen that the sandstone has strong water absorption performance and low longitudinal wave velocity, indicating that the well-developed microscopic pore structure in the Luohe Formation sandstone. To ensure the consistency of mechanical property test, the rock samples were taken from the same piece of parent rock. Besides, the rock samples with large discreteness were removed by wave velocity testing technology in order to reduce the individual differences. Sandstone specimens of Luohe Formation are shown in Fig. 2.

### 2.2 Experimental procedure and device

**Fig. 3:**
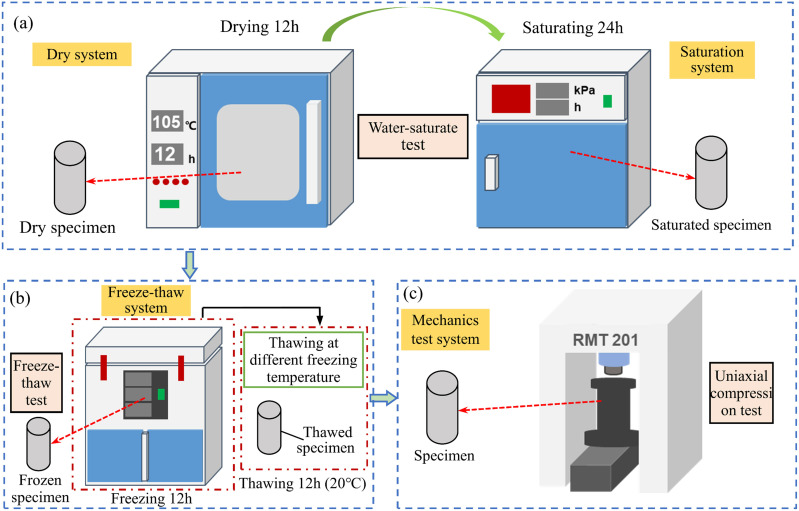
Experimental flow and instrument: **(a)** Intelligent vacuum water saturator instrument and DHG series electric drying oven; **(b)** The multi-function freeze-thaw testing machine; and **(c)** RMT-201 rock and concrete mechanics test system.

Before the freeze-thaw test, Luohe Formation sandstone was placed in a oven box and dried at 105 °C for 12h, then vacuumized in a water-saturated tank until the tank pressure reached -80 kPa, under which the sample was maintained for 6h. The rock samples were continuously soaked for 18h when the negative pressure of the saturation meter rose to 0kPa. The water-saturate test process of rock samples is shown in Fig. 3a. After that, the indoor freeze-thaw tests with different freezing temperatures were carried out when the rock samples were completely saturated. According to the change of the brine temperature curve during the construction of the frozen vertical shaft, the freezing temperatures for freeze-thaw test were set to -30°C, -25°C, -20°C, -15°C and -10°C, respectively. One freeze-thaw test includes freezing the sample at designed freezing temperature in freezer chamber for 12 hours and then thawing the sample at 20°C for another 12 hours. The freeze-thaw test process is shown in Fig. 3b. Finally, the uniaxial compression test of thawed sandstone in Luohe Formation was carried out by using the mechanics test system.

In this study, intelligent concrete vacuum saturation instrument and DHG-1000A series electric drying oven were selected to carry out indoor water-saturate tests, as illustrated in Fig. 3a. In order to study the influence of freeze-thaw on the sandstone in Luohe Formation, the multi-function freeze-thaw testing machine were used to conduct one freeze-thaw cycle test (Fig. 3b). The temperature range of the chamber ranges between -40 and 25°C (a fluctuation of ±0.5°C), as presented in Fig. 3b. In addition, the mechanical properties of sandstone in the Luohe Formation after thawing were investigated by using RMT-201 rock and concrete mechanics test system (Fig. 3c). This test instrument is a computer-controlled electro-hydraulic servo test machine, which consists of an axial loading system, a hydraulic power loading system, a temperature control system and a data acquisition and processing system. The equipment has a maximum axial load of 1500kN, a maximum confining pressure of 50MPa, a loading rate of 0.0001–1 mm/s, and a body stiffness of 6MN/s, as shown in Fig. 3c. The sandstone specimens after thawing were loaded in a displacement-controlled manner, and the loading rate was set to 1×10^-3^ mm/s. The axial pressure was applied to the thawed sandstone until the specimen failure occurs and the mechanical parameters (se.g., deformation and load) were recorded synchronously by the data acquisition system during the loading process.

## 3 Mechanical properties and energy evolution laws

### 3.1 Mechanical characteristics and failure modes of sandstone after thawing

**Fig. 4:**
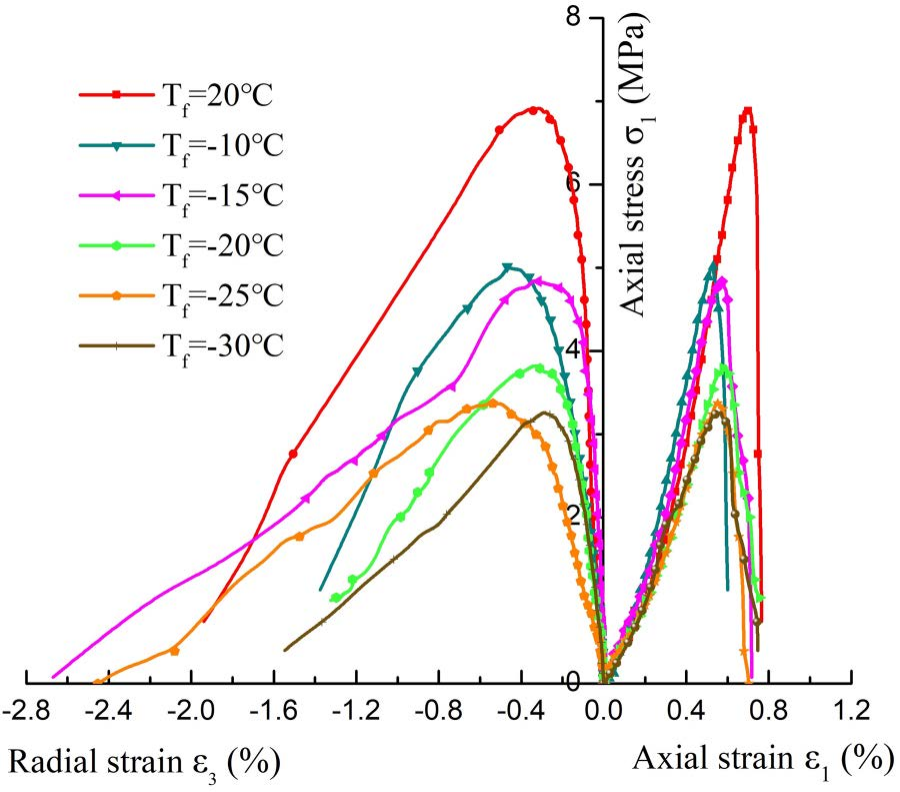
Stress-strain curves of thawed specimen with different freezing temperatures.

The deformation and failure process of Luohe Formation sandstone after thawing presents obvious phased characteristics, and Fig. 4. exhibits the uniaxial compressive stress-strain curves of thawed sandstone at different freezing temperatures. It can be seen from the Fif that the stress-strain curves of thawed sandstone at different freezing temperatures have undergone the pore compaction stage (curve concave), elastic deformation stage (straight line), plastic deformation stage (curve convex), and instability failure stage (curve approximately straight down). The stress-strain curve showed obvious stress drop after the peak point, that is, the rock failure showed obvious brittle failure characteristics (catastrophic failure), and accompanied by a slight cracking sound in the ultimate failure process. Besides, the radial strain of the rock sample is significantly greater than the axial strain during the failure process, indicating that the failure of the sandstone sample exhibits obvious expansion deformation characteristics. Besides, the freezing and thawing temperatures significantly influence the physical and mechanical properties of sandstone [45]. According to the stress-strain curve, table 1 presents the mechanical parameter test results of sandstone after thawing at different freezing temperatures. It can be seen from the table that the compressive strength of thawed sandstone at different freezing temperatures of 20°C, -10°C, -15°C, -20°C, -25°C and -30°C is 6.91MPa, 5.01MPa, 4.85MPa, 3.82MPa, 3.36MPa and 3.26MPa, respectively, indicating the compressive strength decreases by 7.50%, 3.20%, 21.24%, 12.04% and 2.98%. The elastic modulus for different freezing temperatures is 1.19GPa, 0.84GPa, 0.78GPa, 0.71GPa, 0.70GPa and 0.68GPa, respectively, reflecting decreases of 29.41%, 7.14%, 8.97%, 1.41% and 2.86%. The results show that the freeze-thaw process seriously deteriorates the mechanical properties of the Luohe Formation sandstone, resulting in a significant decrease in the strength and deformation parameters of the thawed sandstone. The compressive strength and elastic modulus of the rock samples shows a decreasing trend with the decrease of freezing temperature, which is consistent with the conclusion of Luo’s research [46]. His study pointed out that the influence of freeze-thaw cycles on the mechanical properties of rock is more significant than that of the freezing temperature. On the one hand, the phase transformation from water to ice during the freezing process of saturated sandstone can easily leads to the volume expansion of the micro-pores and micro-fractures in rock interior (the internal volume increases by approximately 9%), creating a large expansion force [47], which result in expanding and then cracking between the weakly cemented sandstone particles. The micro-cracks and micro-fractures in the rock samples continue to develop, expand and connect, resulting in irreversible freezing damage effects in inner sandstone. In addition, the uneven expansion and contraction of mineral components in the sandstone during the freezing-thawing process will induce the internal stress concentration, also lead to the destruction of the internal structure of the sandstone in the Luohe Formatio [48]. On the other hand, The Luohe Formation sandstone contains cementing materials, including unstable and soluble mineral, which are easy to react with water and dissolve in water, making the original pores expand during the process of saturating-freezing-thawing [49]. At the same time, the water-rock interaction under the freeze-thaw process also leads to a decrease in the degree of cementation between sandstone particles. Furthermore, with the continuous decrease of freezing temperature, the freezing damage effect and the water-rock interaction inside the thawed sandstone becomes more and more serious, resulting in strength reduction of the sandstone after thawing. Therefore, the compressive strength and elastic modulus of sandstone after thawing decreases with the decrease of freezing temperature. The peak axial and the radial strain of sandstone after thawing fluctuate with the decrease of freezing temperature, and generally showing a decreasing trend. It is indicated that damage effect caused by one freeze-thaw cycle and water-rock interaction also reduces the deformation-resistant capacity of thawed sandstone, which is consistent with the law of strength weakening of sandstone under the action of single freeze-thaw cycle.

**Table 1.** The mechanical parameters of thawed sandstone under different freezing temperatures.

Freezing temperature (°C)	Peak strength σ_c_ (MPa)	Elastic modulus ^E^ (GPa)	Radial strain of peak point ε_c3_ (%)	Axial strain of peak point ε_c1_ (%)
20	6.91	1.19	0.34	0.70
−10	5.01	0.84	0.46	0.53
−15	4.85	0.78	0.34	0.85
−20	3.82	0.71	0.32	0.58
−25	3.36	0.70	0.55	0.56
−30	3.26	0.68	0.28	0.55

Note: σ_c_ is peak values of the stress-strain curve; E denotes the slope of linear segment in the stress-strain curve; ε_c3_ represents radial strain of peak point in the stress-strain curve; and ε_c1_ represents axial strain of peak point in the stress-strain curve.

**Fig.5.**
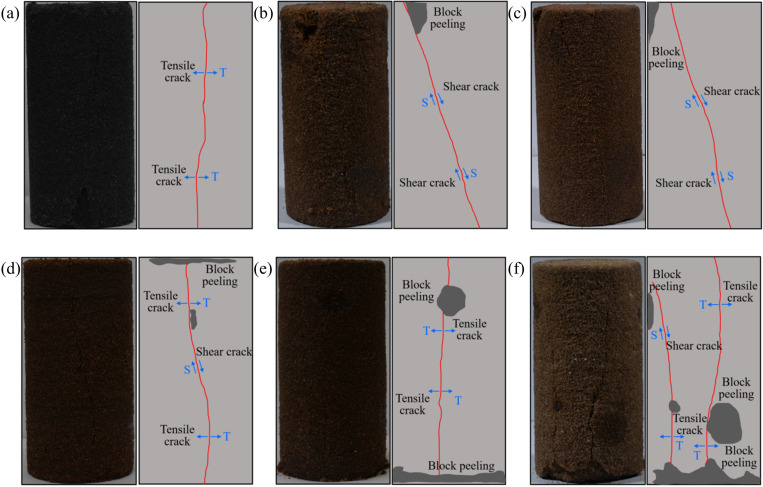
The eventual failure modes of thawed specimens with different freezing temperatures: **(a)** 20°C; **(b)** −10°C; **(c)** −15°C; **(d)** −20°C; **(e)** −25°C; and **(f)** −30°C.

Rock is regarded as porous media, containing initial damage of microcracks and microvoids, which undergo crack closure, initiation, propagation, and coalescence under coupled action of freeze-thaw cycles and uniaxial compression, ultimately leading to instability and failure of rock [50]. The strength weakening of thawed sandstone under freeze-thaw cycles will inevitably influence on its final failure mode, and the final failure mode of thawed sandstone at different thawing temperatures is depicted in Fig. 5. It is observed that the uniaxial compressive failure modes of thawed sandstone are different at different freezing temperatures. Specifically, the tensile cracks penetrate through the sandstone sample in the unfrozen condition, which eventually leads to axial splitting failure of the rock sample (Fig. 5a), which is consistent with other literature [51]. The shear cracks gradually expand to both sides of the rock sample when the freezing temperature is −10°C and −15°C, accompanied with local rock block spalling in the upper part of thawed sandstone and particle shedding on the surface of rock sample, which eventually leads to the unilateral shear failure of the rock sample (Fig. 5b and Fig. 5c). When the freezing temperature is −20°C, the shear cracks in the middle of the rock sample expand to both sides and gradually transform into tensile cracks. Meanwhile, the local rock block spalling occurs at the top and middle of the sample and the surface particle shedding of the rock sample is aggravated, resulting in the tensile-shear mixture failure with tensile cracks (Fig. 5d). The damaged degree of the rock sample is more serious with the decreases of the freezing temperatures. The local rock block peeling and partial fracture occurred in the upper and lower part of the rock sample respectively at freezing temperature of -25°C. Also, the degree of surface particle shedding is further aggravated. The tensile cracks penetrate through thawed rock samples at the same time and ultimately induces tensile failure of the rock sample (Fig. 5e). The tensile crack on the left side of the rock sample expands upwards and gradually transforms into a shear crack when the freezing temperature reduced to −30°C, and the middle tensile crack gradually extends to both sides of thawed sandstone specimen. Moreover, the local rock block spalling occurred in many places of the rock sample, and the most serious partial fracture and particle shedding occurred in the lower part and on the surface of thawed sandstone. The thawed rock sample finally occurred the tensile-shear mixture failure and dominated by tensile cracks (Fig. 5f).

The thawed sandstone mainly has three kinds of destruction patterns under different freezing temperatures, that is, axial splitting failure, shear failure, and tensile-shear mixed failure. The internal structure of the sandstone after thawing will be irreversibly damaged due to the combined influence of freeze-thaw damage and water-rock interaction. Moreover, the weakening of cementation degree between mineral particles decrease the intensity of anti-rupture of thawed rock sample under the load action. Consequently, the surface of Luohe Formation sandstone is accompanied by the local rock block spalling and surface particle shedding after one freeze-thaw cycle. Furthermore, the damage degree inside the thawed sandstone is aggravated with the decrease of freezing temperature, resulting in serious local rock block peeling and surface particle shedding. Meanwhile, the final failure intensity of thawed rock samples observed in uniaxial compression test gradually decrease with the decrease of freezing temperature. Because the phenomenon of rock block spalling and particle shedding in the rock sample after thawing, the elastic strain energy accumulated in the thawed rock sample was released in the form of dissipated energy in advance. Eventually, the elastic strain energy stored in rock sample before the instability and failure is reduced. Consequently, the energy release will inevitably decrease when the rock sample is subjected to catastrophic failure under axial load, resulting in the decrease of failure intensity of the thawed rock sample with the decrease of freezing temperature. A previous study noted that the deterioration due freezing and thawing intensifies with the number of freeze-thaw cycles, resulting in the decrease of tensile crack and the increase of shear crack. In other words, sandstone experiences both tensile and shear failure under uniaxial compression [49]. Furthermore, with the increase of freeze-thaw cycles, the rock surface and internal structure of the sandstone sample becomes loose due to frost swelling pressure, and the particles gradually fall off, eventually resulting in the detachment of a portion of the rock [52]. Those reported conclusions exhibited similar as the local rock block spalling and surface particle shedding under the coupling action of one freeze-thaw cycle and uniaxial compression in this study.

### 3.2 Energy evolution feature of thawed sandstone during deformation and failure

The essence of the deformation and failure of the rock depends on energy accumulation and release, and its final instability and failure is a state instability phenomenon driven by internal energy [53]. Assuming that the physical process is a closed system and there exists no heat exchange with the outside world during the deformation and failure process. According to the first law of thermodynamics (energy conservation theorem), the relationship between the total strain energy of the input rock and the elastic strain energy and the dissipated energy is expressed as [54]:


$$E_{t}=E_{e}+E_{d}$$ (1)

Where *E*_*t*_ denotes the total energy density absorbed by rocks (kJ/m^3^); *E*_*e*_ represents the elastic strain energy density accumulated within the rocks (kJ/m^3^); *E*_*d*_ is the dissipated energy density in the loading process (kJ/m^3^).

In the principal stress space, the total energy density and the elastic strain energy density in the rock can be calculated using Eqs. (2) and (3), as follows:


$$E_{t}=\int_{0}^{\varepsilon_{1}}\sigma_{1}d\varepsilon_{1}+\int_{0}^{\varepsilon_{2}}\sigma_{2}d\varepsilon_{2}+\int_{0}^{\varepsilon_{3}}\sigma_{3}d\varepsilon_{3}$$ (2)

$$E_{e}=\frac{1}{2}\sigma_{1}\varepsilon_{1}^{e}+\frac{1}{2}\sigma_{2}\varepsilon_{2}^{e}+\frac{1}{2}\sigma_{3}\varepsilon_{3}^{e}$$ (3)

where *σ*_*1*_, *σ*_*2*_, and *σ*_*3*_ represent the first, second, and third principal stress, respectively; *ε*_*1*_, *ε*_*2*_, and *ε*_*3*_ represent the first, second, and third principal strain, respectively; $\varepsilon_{1}^{e}$, $\varepsilon_{2}^{e}$, and $\varepsilon_{3}^{e}$ represent elastic parts of the first, second, and third principal strain, which are calculated by Hooke’s theorem.

For the uniaxial compression test, since *σ*_*2*_*=σ*_*3*_=0 in the rock units. The total energy density of the rock sample is calculated using the basic principle of calculus, which is expressed by Eq. (4) as [55]:


$$E_{t}=\int_{0}^{e_{1}}\sigma_{1}d\varepsilon_{1}=\sum_{i=0}^{n}\frac{1}{2}(\sigma_{1i}+\sigma_{1i-1})(\varepsilon_{1i}-\varepsilon_{1i-1})$$ (4)

Where *σ*_*1i*_, *σ*_*1i-1*_, *ε*_*1i*_ and *ε*_*1i-1*_ are the stress and strain values of any two adjacent points in the stress-strain curve; n is the total number of sampling points on the stress-strain curve.

According to the generalized Hooke’s theorem, the calculation formula of elastic strain energy density can be expressed as:


$$E_{e}=\frac{1}{2}\sigma_{1}{\varepsilon_{1}}^{e}=\frac{{\sigma_{1}}^{2}}{2E_{u}}$$ (5)

Where *E*_*u*_ is the elastic modulus of rock unloading, which can be replaced by the initial elastic modulus *E*_*0*_ [56]. Therefore, the elastic strain energy density in the rock can be approximately calculated by use of initial elastic modulus, which is expressed by Eq. (6) as:


$$E_{e}\approx \frac{{\sigma_{1}}^{2}}{2E_{0}}$$ (6)

Therefore, the dissipated energy density during loading can be calculated using Eq. (7):


$$E_{d}=\sum_{i=0}^{n}\frac{1}{2}(\sigma_{1i}+\sigma_{1i-1})(\varepsilon_{1i}-\varepsilon_{1i-1})-\frac{\sigma_{1}^{2}}{2E_{0}}$$ (7)

**Fig. 6:**
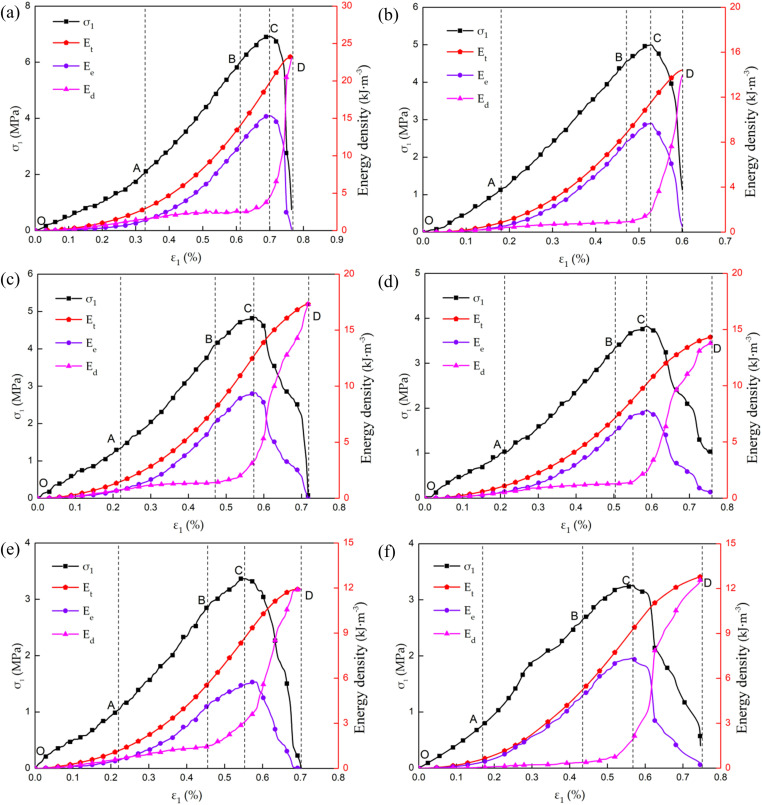
Energy evolution curves of thawed specimens for different freezing temperatures: **(a)** 20°C; **(b)** −10°C; **(c)** −15°C; **(d)** −20°C; **(e)** −25°C; and **(f)** −30°C.

The catastrophic failure of the sandstone in Luohe Formation after thawing is caused by the sudden release of the strain energy accumulated within sandstone, which is closely related to the energy accumulation and dissipation within the rocks. Based on the rock energy dissipation theory, the total energy density, strain energy density and dissipation energy density of sandstone during deformation and failure were calculated by equations (4)-(7). The energy evolution curves of thawed rock samples at different freezing temperatures were obtained, as shown in Fig. 6.

It can be seen from the Fig. 6 that the energy evolution law of sandstone after thawing is basically similar at different freezing temperatures. In the initial compaction stage (section OA), the total energy and elastic strain energy increase nonlinearly, and the dissipated energy also increases nonlinearly due to the closure and friction of micro-defects or micro-cracks in the sandstone, while there is little difference between the elastic strain energy and the dissipated energy. In the elastic deformation stage (section AB), with the gradual closure of the micro-cracks and micro-voids in the rock sample, the increase rate of dissipative energy decreases and the later stage shows a horizontal development trend. There is basically no new dissipated energy produced within the sandstone, and the total energy and elastic strain energy continue to increase nonlinearly at approximately the same rate (concave curve). Meanwhile, the elastic strain energy begins to be greater than the dissipated energy, and the difference between the two kinds of energy continuously increases with the increase of deforming degree of the rock sample. The most of the energy absorbed by rocks is stored within rock sample in the form of elastic strain energy at the elastic deformation stage. In the plastic deformation stage (section BC), the elastic strain energy and total energy continue to increase with the increase of deformation, and the total energy shows a near-linearly increase tendency (the increase rate remains constant) and the elastic strain energy shows a nonlinear increasing trend (the growth rate gradually decreases and closes to zero at the peak point). However, the dissipated energy began to increase and its growth rate gradually increase with the increase of effective deformation, which showed an approximate exponential increasing trend. Because of the nucleation, development and cascade of the micro-defects inside rock sample, and the initiation, propagation, penetration, merging and interaction of the new micro-cracks [57]. As a result, part of the elastic strain energy is dissipated in the form of plastic strain energy and crack surface energy, which resulting in the increase of the dissipation energy rate with the deformation becoming more and more intensive. In the instability failure stage (section CD), the total energy continues to increase, but the growth rate gradually decreases. The elastic strain energy begins to decrease rapidly at a negative growth rate and the dissipated energy increases rapidly at a large growth rate (showing obvious power-law characteristics), and the dissipated energy gradually exceeds the elastic strain energy after the intersection of the two kinds of energy. The elastic strain energy in the rock sample reaches its energy storage limit, the microcracks inside the rock sample begin to expand and penetrate in large quantities and gradually transform into macroscopic cracks, which eventually leads to the instability and failure of the rock sample. Meanwhile, the elastic strain energy stored in the rock sample will be released in the form of kinetic energy, friction energy, and crack surface energy. For freezing-thawing rock samples, the failure is accompanied by obvious local rock spalling and surface particle shedding. The instability and failure of rocks are closely related to the energy accumulation and energy dissipation exhibiting a complex relationship of mutual inhibition and competition [58]. The catastrophic failure exhibiting nonlinearity and irreversibility of the rock sample has been observed when the internal energy release exceeds the energy dissipation.

### 3.3 Temperature effects of thawed sandstone energy evolution

**Fig. 7:**
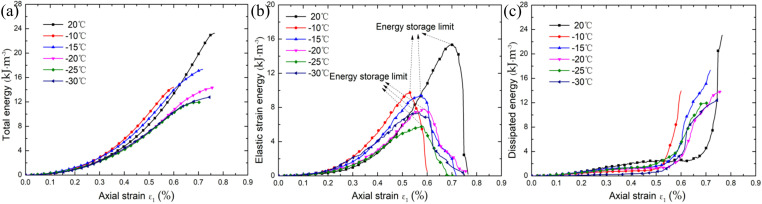
Energy density of thawed specimen under different freezing temperatures: **(a)** Total energy; **(b)** Elastic strain energy; and **(c)** Dissipated energy.

In order to explore the mechanism of energy evolution of sandstone in the Luohe Formation at different freezing temperatures, the analysis focused on the sensitivity of each energy index (i.e., total energy, elastic energy and dissipated energy) to freezing temperature. Fig 7 shows the variation curves of total energy, elastic energy and dissipated energy of thawed rock samples at different freezing temperatures.

The total energy of the sandstone in Luohe Formation after thawing at different freezing temperatures showed an obvious nonlinear increase trend during loading. The total energy increase rate was relatively small at the beginning, then the energy rate increased continuously, and finally gradually slowed down. The total energy evolution curve approximately shows an “S” shape variety rule (Fig. 7a). The total energy growth rate of unfrozen sandstone is the fastest, and total energy absorbed by rocks is the largest during failure. After freezing-thawing, the total energy growth rate and the total energy both decreased during failure, and total energy absorbed by rock sample decreased with the decrease of freezing temperature. Most of the energy input to the rock sample before the peak is stored in the form of elastic strain energy, and a small part is released in the form of dissipated energy. The increase rate of elastic strain energy increases nonlinearly before the peak, gradually decreases when it approaches the energy storage limit, and decreases rapidly after the peak with a large negative growth rate (Fig. 7b). The elastic strain energy stored in the rock sample will be released to the outside in various forms of energy when the rock sample is finally destroyed, and the rate of energy release is significantly affected by the freezing temperature. The elastic strain energy release rate is the fastest when unfrozen sandstone fails (the strain energy curve decreases in a straight line), and the rock failure is the most violent, and the rupture sound is the largest. With the increase of freezing temperature, the downward trend of the strain energy curve after rock failure gradually slows down, indicating that the release rate of elastic strain energy during rock failure decreases with the decrease of freezing temperature, and the intensity of failure also decreases gradually, which once again reveals the internal reason for the decrease of the intensity of rock failure from the energy perspective. The dissipated energy exhibits a non-linear increase, steady development, exponential increase, and rapid increase pattern with the increase of deformation (Fig. 7c). The increase rate of energy dissipation during rock failure is also significantly affected by freezing temperature. With the decrease of freezing temperature, the increase rate of dissipated energy and the slope of the dissipated energy evolution curve decrease, which is consistent with the law of elastic strain energy release. It is indicates that the freezing-thawing process significantly reduces the intensity of rock failure. Therefore, the intensity of rock failure is closely related to the release rate of strain energy and the increase rate of dissipated energy. The strain energy release rate and the dissipation energy increase rate of rock samples generally decrease with the decrease of freezing temperature, which corresponds to the intensity and failure mode of rock samples mentioned in section 2.1 (Fig. 5).

When the elastic strain energy accumulated in the rock sample reaches its energy storage limit, the catastrophic failure of the rock can be carried out spontaneously without applying the external work increment continuously, that is, the “energy self-sustaining process”. Therefore, it is difficult to reverse and control the catastrophic failure and a small increment of the control variable (e.g., displacement) will cause a finite response (e.g., energy) of the rock [59]. In order to study the influence of each energy index of the peak point by different thawing temperatures, the energy index values of the peak point after sandstone thawing under different freezing temperatures are given in Table 2. It can be seen that the proportion of peak point elastic energy after sandstone thawing at different temperatures is 72.35%, 83.30%, 72.44%, 76.19%, 63.19%, 63.68% and 79.31%, respectively, and the proportion of dissipated energy is 27.65%, 16.70%, 27.56%, 23.81%, 36.32% and 20.69%, respectively. The results indicate that the energy conversion before the peak of sandstone is mainly the storage of elastic strain energy. A small part of the strain energy stored in the rock sample is released in the form of dissipated energy due to the closure friction of microcracks and micropores, the initiation and expansion of new microcracks and the fragmentation of mineral crystals in the Luohe Formation sandstone. Most of the energy of the input rock samples is stored in the mineral lattice in the form of elastic strain energy due to the elastic deformation of the mineral crystals. So the total energy of the input rock samples is mainly converted into elastic strain energy, and the proportion of dissipated energy is relatively small.

**Table 2.** The energy density of peak point with different freezing temperatures.

Freezing temperature(°C)	Total energy (kJ·m^-3^)	Elastic strain energy(kJ·m^-3^)	Elastic energy proportion (%)	Dissipated energy(kJ·m^-3^)	Dissipated energy proportion(%)
−20	20.15	14.58	72.35	5.57	27.65
−10	11.68	9.73	83.30	1.95	16.70
−15	13.03	9.44	72.44	3.59	27.56
−20	10.25	7.81	76.19	2.44	23.81
−25	8.84	5.63	63.68	3.21	36.32
−25	8.84	5.63	63.68	3.21	36.32
−30	9.33	7.40	79.31	1.93	20.69

**Fig. 8:**
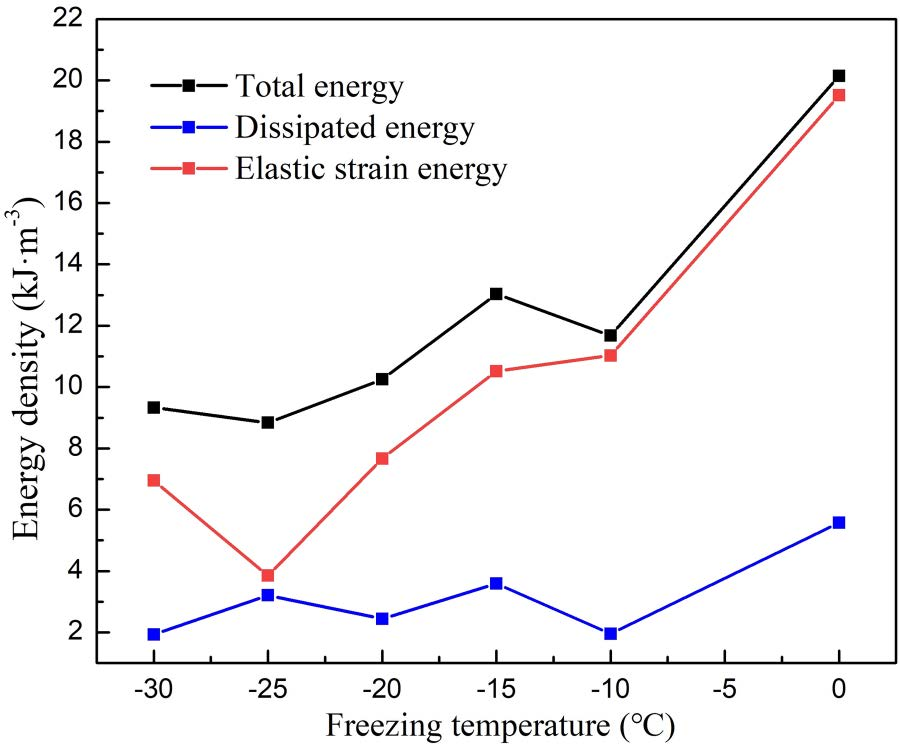
The variation curve of the energy density at the peak point with different freezing temperature.

Based on the data in Table 2, the variation of peak point energy index with freezing temperature after sandstone thawing can be obtained, as shown in Fig 8. It can be seen from the diagram that the total energy at the peak point of sandstone at different temperatures is 20.15 kJ·m^-3^, 11.68 kJ·m^-3^, 13.03 kJ·m^-3^, 10.25 kJ·m^-3^, 8.84 kJ·m^-3^ and 9.33 kJ·m^-3^, respectively, and the elastic strain energy is 14.58 kJ·m^-3^, 9.73 kJ·m^-3^, 7.44 kJ·m^-3^, 7.81 kJ·m^-3^, 5.63 kJ·m^-3^ and 7.40 kJ·m^-3^, respectively. Elastic strain energy and total energy fluctuate with the decrease of freezing temperature, generally showing a decreasing trend. The elastic strain energy at the peak point characterizes the energy storage limit of the rock mass, and the larger the energy storage limit, the less susceptible the rock is to energy-driven failure. With the decrease of freezing temperature, the degree of freezing damage of sandstone is aggravated, and the interaction between water and rock is enhanced after thawing, which leads to the weakening of the ability of sandstone to resist deformation and failure. The small energy input into the rock sample will make it reach the energy storage limit and enter the energy self-sustaining process, hence the elastic strain energy and total energy at the peak point decrease with the decrease of freezing temperature. In addition, the dissipated energy at the peak point also gradually decreases with the decrease of freezing temperature, but the change amplitude is very small compared with the total energy and elastic energy. The degree of freezing damage will be aggravated and the bearing performance of the rock sample will been weaken with the decrease of freezing temperature, resulting in the continuous reduction of the input energy and the elastic strain energy stored in the rock. Therefore, the energy released in form of the dissipated energy will also decrease accordingly when there are new cracks in the rock under the action of load.

## 4 Critical power-law catastrophe failure prediction theory

When the material approaches the catastrophic failure point, the sensitivity of response variable to the response of external control variables will significantly increase, which is called critical power-law singularity [60,61]. The critical power-law singularity is a typical precursory feature of material catastrophic failure, which can be used to carry out the research on catastrophic failure prediction after thawing of the sandstone in Luohe Formation. The two key factors affecting the prediction results of catastrophe are the acquisition of critical power-law characteristic test data and the establishment of failure prediction methods.

### 4.1 Energy damage variables of thawed sandstone

Energy accumulation and dissipation, as the driving force behind the nucleation of mesoscopic cracks in rocks, will inevitably lead to irreversible damage effects on rocks, resulting in weakening of rock strength and instability. In the process of damage and destruction, the rock always converts energy and matter with the outside world in different forms, in which the energy input from the outside is mainly converted into elastic energy and dissipative energy [62]. The energy dissipation, as the essential attribute of rock deformation and failure, is the process of continuous development, extension, derivation, and weakening of the internal structure of the rock [63]. Therefore, the energy dissipation reflects the attenuation of the rock strength to a certain extent, and the energy damage variable D can be defined by using the dissipative energy as [64]:


$$D=\frac{E_{d}}{E_{dc}}$$ (8)

where *E*_*dc*_ is the critical dissipative energy (kJ/m^3^) when the unit strength is lost, that is, the dissipated energy corresponding to the peak point.

### 4.2 Critical power-law singularity

Critical power-law singularity is a typical precursory feature of material catastrophic failure, that is, the small change of the response variable relative to the control variable will accelerate the development, showing the precursory characteristics of critical power-law singularity prior to material failure phenomena [65].


$$\frac{d\Omega }{d\lambda }=k{\left(\lambda_{f}-\lambda \right)}^{-\beta }$$ (9)

where *Ω* is the observable response variable of rock deformation and failure, -*β* is the critical power-law singularity index (critical singularity characteristic value), *k* is the proportionality constant, *λ* is the control variable (such as displacement or force), *λ*_*f*_ is the control variable of imminent rock failure, and *dΩ/dλ* is the change of the response variable relative to the control variable [66].

In general, the loading control variable and the time perfectly conform to linear relationship, so the above equation (9) can be rewritten as the form of a power-law increase in the rate of precursory signals with time (accelerated evolution rate of the response variable) [67]:


$$\frac{d\Omega }{dt}=k{\left(t_{f}-t\right)}^{-\beta }$$ (10)

where *t* represents the evolution time of the control variable, *t*_*f*_ denotes the time of imminent rock failure, and *dΩ/dt* denotes the change rate of the response variable Ω.

The energy damage variable defined by dissipative energy can characterize the essential properties of rock deformation and failure, and the energy damage variable can be used as the response variable to analyze the critical singularity characteristics of thawed Luohe Formation sandstone under uniaxial compression with different freezing temperatures. The critical accelerated failure process of energy damage variable D can be described as:


$$\frac{dD}{dt}=k{\left(t_{f}-t\right)}^{-\beta }$$ (11)

where dD/d*t* represents change rate of the energy damage variable (energy damage variable rate).

Take both sides of the equation to the power of *-1/β* at the same time, and rewrite (11) as:


$${\left(\frac{dD}{dt}\right)}^{-\frac{1}{\beta }}=k^{-\frac{1}{\beta }}(t_{f}-t)$$ (12)

From the above equation, it can be seen that *(dD/dt)*^*-1/β*^ has a good linear relationship with the time *t*, and the energy damage variable rate shows critical power-law singularity behavior when the critical failure of Luohe Formation sandstone occurs after thawing. Consequently, *(dD/dt)*^*-1/β*^ tends to 0 when the thawed sandstone tends to the failure point.

In order to intuitively describe the critical power-law characteristics of energy damage variables rate, take both sides of Eq. (11) to the logarithm and transform it to:


$$\log_{10}(\frac{dD}{dt})=\log_{10}k-\beta \log_{10}(t_{f}-t)$$ (13)

It can be seen that Eq. (13) presents a linear relationship between the change rate of the energy damage variable and the time with a slope of *-β*. Therefore, the failure time of sandstone in Luohe Formation after thawing can be analyzed and the corresponding catastrophic failure early warning can be carried out according to the power-law singularity characteristics of response variable of imminent rock failure

### 4.3 Cataclysmic failure prediction method

The critical power-law acceleration process of the response variable during rock failure is essentially a dynamic process induced by the internal damage evolution, which leads to the obvious abnormal behavior characteristics of the response variable before the failure. This acceleration feature is the precursory signal for identifying and predicting the catastrophic failure of the rock, so the critical power-law singularity feature of the response variable can be used to forecast the rock failure time. It is worth noting that, however, the critical power-law singularity index of the response variable exhibits a considerable scatter (large dispersion) in practice. The power law exponent is affected by to rock material, response variable, loading method, loading condition and other factors in the field measurement [68,69]. Because the value of the critical power-law singularity index cannot be measured and determined before rock failure, it is necessary to explore the failure time prediction of rock under the condition of unknown critical power-law singularity index.

The critical acceleration process of the energy damage variable rate before the strength failure of thawed sandstone in the uniaxial compression test can be expressed by the power law relation (11), and there is a certain discreteness in the power exponential *β*. In order to explore the failure time forecast method based on the precursory characteristics of critical power-law singularity of imminent rock failure, the derivative of t on both sides of the critical power-law relation (11) is obtained (differentiate with respect to *t* on both sides of the critical power-law relation (11):


$$\frac{d^{2}D}{dt^{2}}=\beta k{\left(t_{f}-t\right)}^{-\beta -1}$$ (14)

where $\frac{d^{2}D}{dt^{2}}$ represents the acceleration of the energy damage variable (energy damage variable acceleration).

Take *(β+1)/β* power on both sides of equation (14) at the same time:


$${\left(\frac{dD}{dt}\right)}^{\frac{\beta +1}{\beta }}=k^{\frac{\beta +1}{\beta }}{\left(t_{f}-t\right)}^{-\beta -1}$$ (15)

Combined formula (12) and (14):


$$\frac{d^{2}D}{dt^{2}}{\left(\frac{dD}{dt}\right)}^{-\frac{\beta +1}{\beta }}=\beta k^{-\frac{1}{\beta }}$$ (16)

Further simplified Eq. (16):


$$\frac{d^{2}D}{dt^{2}}{\left(\frac{dD}{dt}\right)}^{-\alpha }=A$$ (17)

or


$$\ddot{D}{\dot{D}}^{-\alpha }=A$$ (18)

Among them, the index:


$$\alpha =1+\frac{1}{\beta }$$ (19)

where the constants *A=βk*^*(-1/β)*^, $\frac{dD}{dt}$ = $\dot{D}$ and $\frac{d^{2}D}{dt^{2}}$ = $\ddot{D}$ are the first and second derivatives of the energy damage variable to time t, respectively, and Eq. (18) is the empirical relation given by Voight [70].

Order: $\dot{D}$ =y, $\ddot{D}$ = $\dot{y}$, and bringing it into the equation (18):


$$\dot{y}y^{\alpha }=A$$ (20)

Integrating both sides of equation (20):


$$\frac{1}{1-\alpha }y^{1-\alpha }=At+C$$ (21)

where C is a constant, when *t* tends to *t*_*f*_, $\dot{D}$ =0, so that C=*-At*_*f*_, bringing it into Eq. (21) above:


$$\frac{1}{1-\alpha }y^{1-\alpha }=-A(t_{f}-t)$$ (22)

Term-shifting and take the power of 1/(1-α) on both sides:


$$y=\dot{D}=\frac{dD}{dt}={\left[A\left(\alpha -1\right)\right]}^{\frac{1}{1-\alpha }}{\left(t_{f}-t\right)}^{\frac{1}{1-\alpha }}$$ (23)

Take the logarithm on both sides of Eq. (23):


$$\ln\dot{D}=\frac{1}{1-\alpha }\ln\left[A\left(\alpha -1\right)\right]+\frac{1}{1-\alpha }\ln(t_{f}-t)$$ (24)

Take the logarithm on both sides of equation (18), which is:


$$\log\ddot{D}=\alpha log\dot{D}+\log A$$ (25)

From the above equation, it can be seen that there is a good linear relationship between the logarithm of the energy damage variable acceleration and logarithm of the energy damage variable rate. In practical application, the power-law singularity trend of response variable of imminent rock failure has been routinely used to forecast the failure time. Specifically, the energy damage variable D obtained by monitoring is used to calculate the energy damage variable rate $\frac{dD}{dt}$ and energy damage variable acceleration $\frac{d^{2}D}{dt^{2}}$; and then the α is obtained by linear fitting according to the relationship between $\frac{d^{2}D}{dt^{2}}$ and $\frac{dD}{dt}$ in equation (25); then the critical power-law singularity index β is calculated by equation (19); and finally based on the linear relationship between ($\frac{dD}{dt}$)^-1/β^ and time *t*, the catastrophic failure time of the rock is predicted according to the linear extrapolation method in equation (12).

**Fig. 9:**
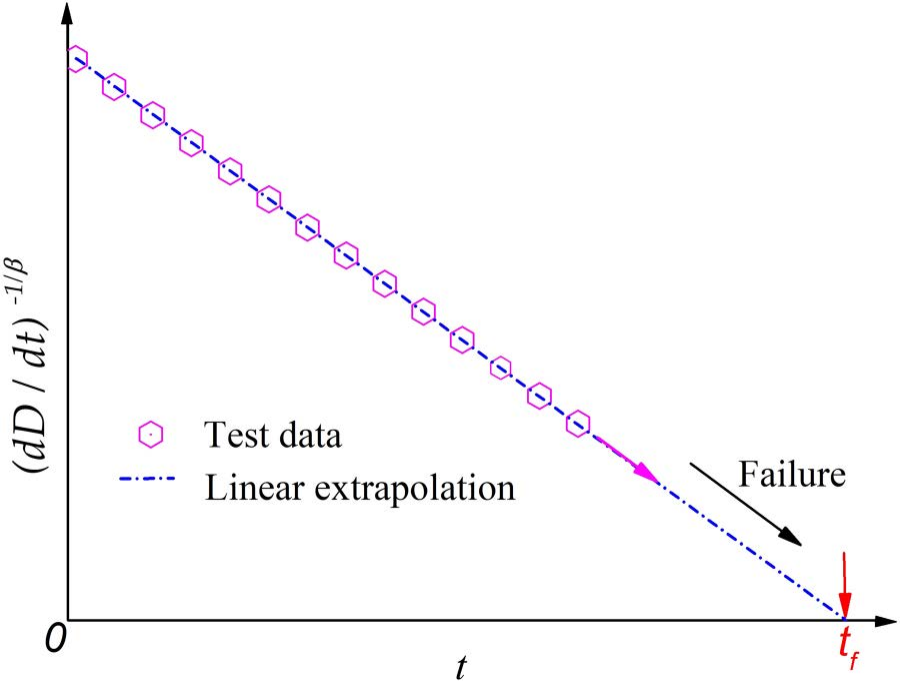
Sketch map of predicted failure time through linear extrapolation.

The monotonic variation of slope lays a foundation for predicting rock catastrophic failure based on the critical power-law singularity exponential range. It can be seen from equation (12) that ($\frac{dD}{dt}$)^-1/β^ is linearly related to time t, and it tends to zero when the rock fails. Thus, the failure time forecast of the rock can be carried out. The rock has formed a macroscopic penetration crack (rock instability failure) and the rock failure time *t*_*f*_ can be deduced when the value of ($\frac{dD}{dt}$)^-1/β^ reaches the zero. The least squares method is used to fit the test data, and the oblique line is linearly extrapolated to the abscissa axis with ($\frac{dD}{dt}$)^-1/β^=0. The predicted failure time can be determined as the intersection point of the oblique line and the transverse axis, and the prediction effect will become more and more obvious with the gradual increase of monitoring data. Sketch map of predicting failure time through linear extrapolation is depicted in Fig. 9.

## 5 Critical power-law singularity and exponential of thawed sandstone

### 5.1 Evolution characteristics of energy damage response

**Fig. 10:**
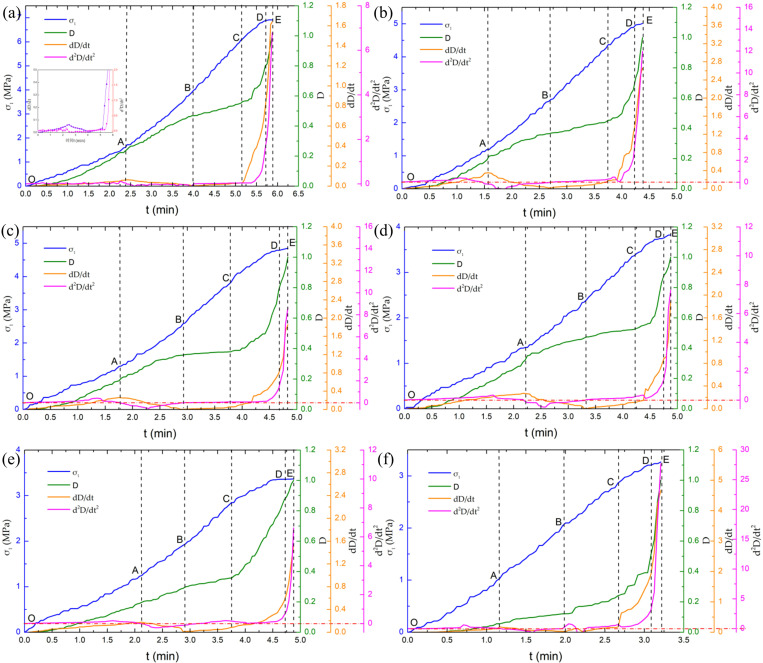
Energy damage variable, energy damage variable rate and acceleration of thawed sandstone with different freezing temperatures: **(a)** 20°C; **(b)** -10°C; **(c)** -15°C; **(d)** -20°C; **(e)** -25°C; and **(f)** -30°C.

There is a very important correlation between the catastrophic failure characteristics and the response variable when the material was close to failure. The sensitivity of the system response variable to the control variable is significantly increased (i.e., critical sensitivity) when the system tends to catastrophic failure. The change rate of the response variable to the control variable is a response function, and a small increment of the control variable before the catastrophic destruction will cause a sharp change in the response variable. In order to explore the variation law of the energy damage response variables after sandstone thawing at different freezing temperatures, the response variable *D* was calculated according to the formula of energy damage variables, and the change rate of damage variables *dD/dt* and the acceleration of damage variables *d*^*2*^*D/dt*^*2*^ with time were obtained. According to the definition of damage variables, analyzing the data before the peak because the damage variable is equal to 1 at the peak point, and then the value is greater than 1 and continues to increase. Finally, the pre-peak damage variables rate and acceleration evolution curves of the Luohe Formation sandstone after thawing are given at different freezing temperatures, as presented in Fig. 10.

It can be observed that the energy damage variable rate of the Luohe Formation sandstone in the whole deformation process undergoes a nonlinear increase, peak, approximate linear decrease, trough, approximate linear increase, acceleration increase and sharp increase stage, and the catastrophic failure of the sandstone shows obvious critical power-law singularity characteristics from the accelerated increase to the sharp increase stage. The occurrence of internal damage (microcracks, micropores, macroscopic cracks, etc.) in the process of deformation of rock samples will inevitably cause the elastic strain energy accumulated in the rock to be released in the form of dissipated energy. The energy damage variable defined by dissipated energy can be used to characterize the damage degree within rock, and the corresponding evolution of the energy damage variable rate shows obvious stage characteristics. Therefore, based on the evolution characteristics of the energy damage variable rate combined with the change law of energy damage variable and the energy damage variables acceleration, the evolution of energy damage of thawed sandstone under uniaxial compression can be divided into five stages.

(1) Minor damage stage (OA section), that is, the increases stage of energy damage variable rate. In this phase, the growth rate of the damage variable is small in the early stage, and the growth rate gradually increases in the later stage. the curve of damage variable shows a nonlinear increasing trend (concave), which leads to an approximate linear increase of the energy damage variable rate in the early stage. It indicates that the damage inside the rock sample is constantly developing, which is mainly due to the stress adjustment inside the rock sample, resulting in the energy absorbed by rocks is mostly used for friction between the mineral lattice and closure of the microcracks and micropores inside the rock sample. Minor damage was produced inside the rock sample. The energy dissipation in the rock sample gradually decreases with the continuous closure of microcracks and micropores, and the energy damage variable rate curve gradually slows down in the later stage. Subsequently, the internal damage rate of the rock sample reaches the maximum value at this stage (the slope of the damage variable curve is the largest) when it reaches peak. The corresponding energy damage variable acceleration basically remains horizontal in the early stage and gradually decreases in the later stage. After the energy damage variable rate reaches the first small peak, the energy damage evolution of the sandstone in Luohe Formation enters the deceleration damage stage.
(2) Deceleration damage stage (AB section), that is, the decreases stage of energy damage variable rate. The curve of the energy damage variable is concave at this stage. the energy damage variables rate decreases linearly with a relatively fast rate in the early stage and a gradual slowdown in the later stage, indicating that the internal damage rate of the rock sample gradually slows down. The energy damage variable acceleration decreases continuously, and then gradually increase after reaching the minimum value. Due to the closed microcracks and micropores in the rock sample, the sandstone mineral crystals have undergone elastic deformation. Most of the input energy being stored in the mineral lattice in the form of elastic strain energy, and part of the input energy is dissipated in the internal microcrack friction of the rock sample and friction between mineral particles. In the slight injury stage and the deceleration injury stage, the energy damage variable rate and the acceleration of the energy damage variable slowed down with time in an inverted “V” shape. There were two peaks in this process (Fig. 10a), in which the peak acceleration of the energy damage variable was ahead of the peak value of the energy damage variable rate. When the acceleration of the energy damage variable peaks, the external influence on the internal damage rate of the rock reaches the maximum, that is, the closure and friction of the microcracks and micropores in the rock are the largest. When the energy damage variable rate peaks, the internal damage rate of the rock reaches the maximum value. In the minor damage stage and the deceleration damage stage, the energy damage variable rate and the energy damage variable acceleration slowed down with time in an inverted “V” shape (Fig. 10a), in which the peak value of the energy damage variable acceleration was ahead of the peak value of the energy damage variable rate. When the energy damage variable acceleration reaches the peak, the influence of the external force on the internal damage rate of the rock reaches the maximum, that is, the closure effect and friction effect of the microcracks and micropores in the rock are the largest. when the energy damage variable rate reaches the peak, the internal damage rate of the rock reaches the maximum value.
(3) Damage steadily increases stage (BC section), that is, the stable stage of energy damage rate. The evolution of energy damage begins to enter the damage steadily increase stage after the energy damage variable rate reaches a low point. At this stage, the energy damage variable rate curve increases linearly with a slight slope, indicating that the damage rate in the rock sample increases gradually. The curve of the energy damage variable shows a smooth nonlinear increasing trend and its slope increases gradually, while the energy damage variables acceleration is roughly horizontal because the curve of the energy damage variable rate increases approximately linearly with time. Because the microcracks or micropores inside rock sample have been completely closed, the elastic strain energy stored in the mineral lattice gradually increases. When the elastic strain energy of some mineral lattices exceeds its energy storage limit, gradual fractures between mineral particles in the sandstone of the Luohe Formation and the formation of new micro-cracks occur. The generation of new micro-cracks is closely related to the deformation and failure of micropores and microcracks, while the number of new micro-cracks inside the sandstone keeps increasing at a small rate. The increase in the number of microcracks aggravate the damage rate in the rock sample, so the energy damage variable rate curve shows a slight linear increasing trend. On the whole, the cumulative damage degree in the sandstone at this stage is relatively small.
(4) Damage accelerated increase stage (CD section), that is, the rapid growth stage of energy damage variable rate. As the Luohe Formation sandstone is continuously compressed, more and more mineral lattices begin to exceed its energy storage limit, resulting in the internal microdefects of the rock sample to continuously damage nucleation, development and cascade. A localized zone developed by a large number of micro-damages and micro-cracks was gradually developed in the rock sample, and most of the elastic strain energy began to be dissipated by the localized zone in the form of plastic strain energy and micro-crack surface energy. The energy release and energy dissipation were basically in equilibrium state. Furthermore, the internal new microcracks increase at a significant rate due to the continuous development and evolution of the localized zone, resulting in rapid energy dissipation in the rock samples. The energy damage variable increases with time, and the energy damage variable rate and the energy damage variable increase significantly compared with the previous stage, showing an exponential increasing trend. The slopes of the three curves are as follows: the energy damage variables acceleration> the energy damage variables rate> energy damage variables.
(5) Damage rapid increase stage (DE segment), that is, the sudden increase stage of energy damage variable rate or catastrophic destruction stage. The catastrophic failure depends on the competing relationship between the energy dissipation in the localized area and the energy release in the non-localized area, which is closely related to the localized area scale and the damage evolution characteristics of the localized area. At the critical peak point (at the time of failure), the continuous development and evolution of the damage and deformation of the localized zone is driven by the energy release of the non-localized zone in the rock sample, and the localized zone gradually expands, extends and connects with each other. The sudden catastrophic failure of the rock sample will occur when the energy release of the non-localized zone exceeds the energy dissipation capacity of the localized zone [71]. At this stage, the cascade and interaction of the localized zone in the rock sample caused the macroscopic cracks of the rock sample to expand and penetrate, forming a macroscopic fracture surface that pass through the rock sample (Fig. 5). The elastic strain energy stored in the rock sample is rapidly released in the form of crack surface energy, and the release of elastic strain energy exceeds the ability of the energy dissipation in the localized zone, and the remaining energy is converted into kinetic energy and friction energy of the rock sample failure. Due to the energy dissipated in the form of kinetic energy, friction energy and crack surface energy, the curve of the energy damage variable, the energy damage variable rate and the energy damage variable acceleration increase sharply and show obvious critical power-law singularity. Moreover, the growth rate of the energy damage variable acceleration curve is greater than the energy damage variable rate curve, indicating that the rock sample is in the catastrophic failure stage with the continuous increase of energy damage acceleration, and the damage acceleration represents the essential attributes of rock catastrophic failure. The formation of macroscopic fracture planes eventually leads to the lose of the bearing capacity and the occurrence of unstable failure of rock samples. A small time increment at this stage will cause the change rate of the response amount (energy damage variable) relative to the control amount (time) to tend to be infinite, that is, it will generate singularity. Therefore, the energy damage variable rate will show the accelerated development characteristics when the Luohe Formation sandstone is close to catastrophic failure after thawing, and the critical acceleration process presents obvious power-law singularity acceleration characteristics [72].

The rocks were composed of a variety of mineral grains, weak cements and developed pore defects, and the random spatial distribution of micro-damage, micro-defects and micro-cracks leads to slight differences in the evolution curve of energy damage after sandstone thawing. However, the evolution process of the energy damage of the Luohe Formation sandstone after thawing at different freezing temperatures goes through five stages: minor damage stage, deceleration damage stage, damage steadily increase stage, damage accelerated increase stage, and damage sharply increase stage (catastrophic failure stage). It’s worth noting that the change rate of energy damage variable is exponentially related to time in the stage of accelerated damage increase, that is, the energy damage rate tends to increase in the form of exponential function. In the final catastrophic failure stage, deformation and damage lead to the formation of a continuous localized area (continuous damage area) in the Luohe Formation sandstone, and the localized area develops rapidly until the sample is unstable and failure [73]. The acceleration evolution law of the energy damage variables, the energy damage variable rate and the energy damage variables acceleration all show obvious critical power-law singularity behaviors.

### 5.2 Critical singularity characteristics of thawed sandstone

**Fig. 11:**
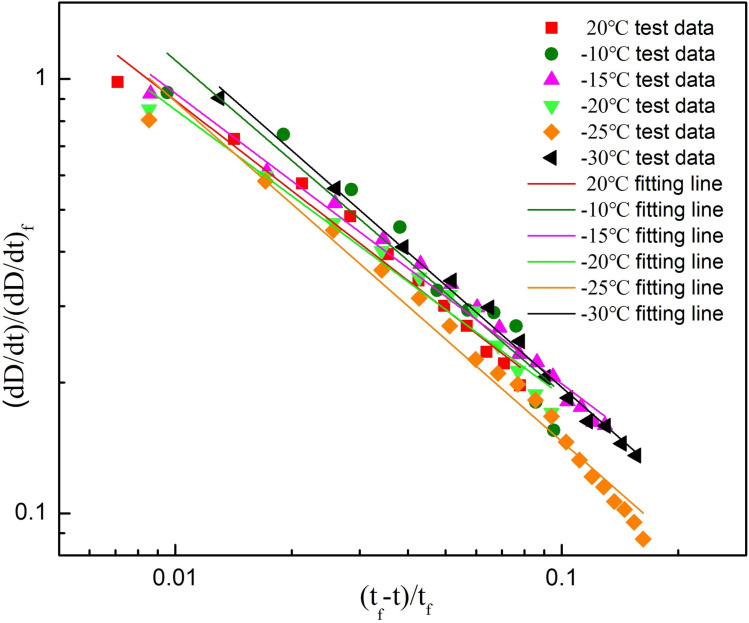
Double-logarithm fitting plots of energy damage rate and time of thawed sandstone at different freezing temperatures.

The critical power-law singularity of the response function (energy damage variable rate) is a typical precursory feature of rock catastrophic failure, and it is very important to understand the catastrophic failure mechanism of the Luohe Formation sandstone after thawing. Based on the variation of the energy damage variables rate in the Luohe Formation sandstone, the experimental data of the area near the critical failure point are processed by using the above-mentioned critical power-law catastrophe failure prediction theory (Section 3), and the change rate of energy damage variables *(dD/dt)* are then obtained. Simultaneously, the *(dD/dt)/(dD/dt)*_*f*_ and *(t*_*f*_*-t)/t*_*f*_ are normalized. In order to observe the critical power-law phenomenon of the deformation and failure process of sandstone after thawing, and to explore the critical power-law singularity as a catastrophic failure precursory signal, the horizontal and longitudinal coordinates of the relationship between *(dD/dt)/(dD/dt)*_*f*_ and *(t*_*f*_*-t)/t*_*f*_ were processed by double logarithmic processing, and the linear fitting of the double logarithmic graph was carried out by the least squares method. Finally, Double-logarithm fitting plots of energy damage variable rate and time of thawed Luohe Formation sandstone at different freezing temperatures are presented in Fig. 11.

It can be seen from Fig. 11 that the double logarithmic diagram of the energy damage variables rate and time is approximately linear relationship during the energy damage accelerated development of the Luohe Formation sandstone after thawing at different freezing temperatures. Among them, the slope of the fitting line in the double logarithmic diagram is the critical power-law singularity index β of the thawed sandstone, which indicates that the critical acceleration phenomenon of the energy damage variable rate only occurs when the rock specimen is approaching to failure. After obtaining the double logarithmic and normalization of energy damage variables rate and time, the thawed rock samples presented a critical precursory behavior of linear evolution when the damage variable approaches the rock structure destruction level. The slope of fitting straight line in Fif 11 is presented in Table 3.

**Table 3.** Power law singularity index (β) of thawed sandstone under different freezing temperatures.

Freezing temperatures	0	−10	−15	−20	−25	−30	Average value
β	0.68	0.76	0.66	0.65	0.78	0.78	0.72

It can be seen from the diagram that the critical power-law singularity indices corresponding to energy damage variables change rate of thawed sandstone at different freezing temperatures has a certain degree of dispersion, but the degree of dispersion is small, and there are crossings between the fitting lines with a relatively small intersection angle. The critical power-law singularity indices after sandstone thawing at different freezing temperatures (0, −10, −15, −20, −25 and −30°C) were 0.68, 0.76, 0.66, 0.65, 0.78 and 0.78, respectively. It can be concluded that the Luohe Formation sandstone exhibits obvious critical singularity evolution characteristics during catastrophic failure after thawing, and the corresponding critical power-law singularity characteristic β range is roughly 0.7±0.1. The critical power-law acceleration precursory characteristics of the energy damage variable change rate of the thawed Luohe Formation sandstone are closely related to the catastrophic failure, showing a typical linear relationship which provides the possibility and convenience for the catastrophic failure prediction of the Luohe Formation sandstone after thawing.

### 5.3 Catastrophic failure time prediction of thawed sandstone

**Fig. 12:**
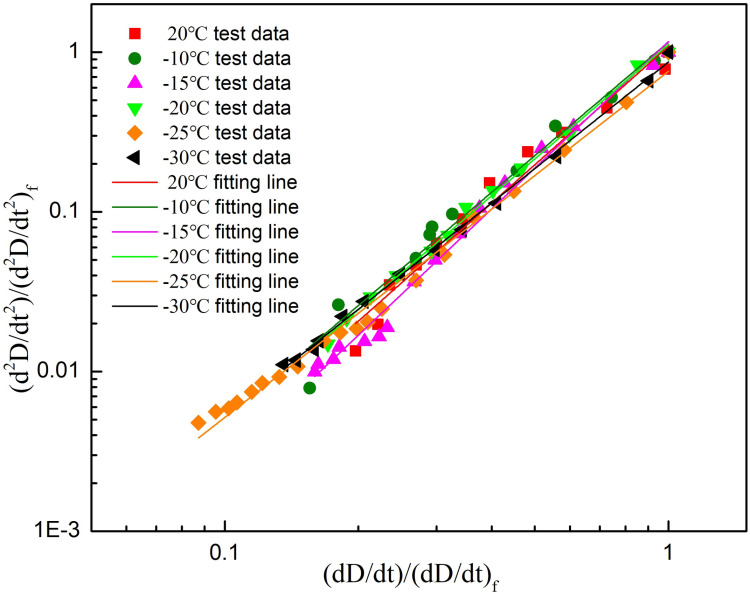
Double-logarithm fitting plots of energy damage rate and acceleration of thawed sandstone with different freezing temperatures.

The damage variable rate exhibits the power-law acceleration characteristics when the rock sample was close to failure, and the failure time of sandstone samples can be predicted by using this precursory feature. However, due to the discreteness of the power-law singularity index and the inability to determine the specific value before failure, the catastrophic prediction method mentioned above (Section 4.3) can be used to predict the failure time of rocks under the unknown critical power-law singularity index. By processing and calculating the monitoring data, the energy damage variable change rate and its acceleration in the critical failure stage of sandstone were obtained. Meanwhile, the data of energy damage acceleration and its change rate were normalized, and the double logarithmic curves of energy damage acceleration and change rate were fitted by the least squares method. Finally, the double-logarithm fitting straight line of energy damage variable rate and the acceleration of thawed sandstone with different freezing temperatures were presented in Fig. 12. Combined with equation (25), it can be seen that the slope of the fitted straight line in Fig.12 is *α*, and then the critical power law singularity index value *β*_*1*_ after sandstone thawing can be calculated from *α*=1+1/*β*_*1*_. The slope value α of the straight line and the power index value *β*_*1*_ are given in Table 4. Ultimately, based on the linear extrapolation failure time prediction method (Fig. 9), the catastrophic failure time of the thawed Luohe Formation sandstone under different freezing temperatures is predicted, and the resulting failure time is the predicted value of catastrophic failure time (*t*_*f*_^*p*^).

**Table 4.** Power law singularity index of (β_1_) thawed sandstone under unknown β value conditions.

Freezing temperatures	0	−10	−15	−20	−25	−30
α	2.46	2.35	2.62	2.37	2.16	2.21
β_1_	0.68	0.74	0.61	0.72	0.86	0.82

Comparing the critical power-law singularity indices in Table 3 and Table 4, the power exponents obtained by the catastrophic failure time prediction method in Section 4.3, are very close to those obtained based on the change rates of the damage variables when the critical power-law singularity index is unknown. The absolute values of the power exponents at different freezing temperatures (0, −10, −15, −20, −25 and −30°C) are 0, 0.02, 0.05, 0.07, 0.08 and 0.04, respectively, and the maximum absolute difference is 0.08. Therefore, based on the proximity failure monitoring data, the critical power-law singularity index calculated by using the established catastrophic failure time prediction method fell within a reasonable range.

**Fig. 13:**
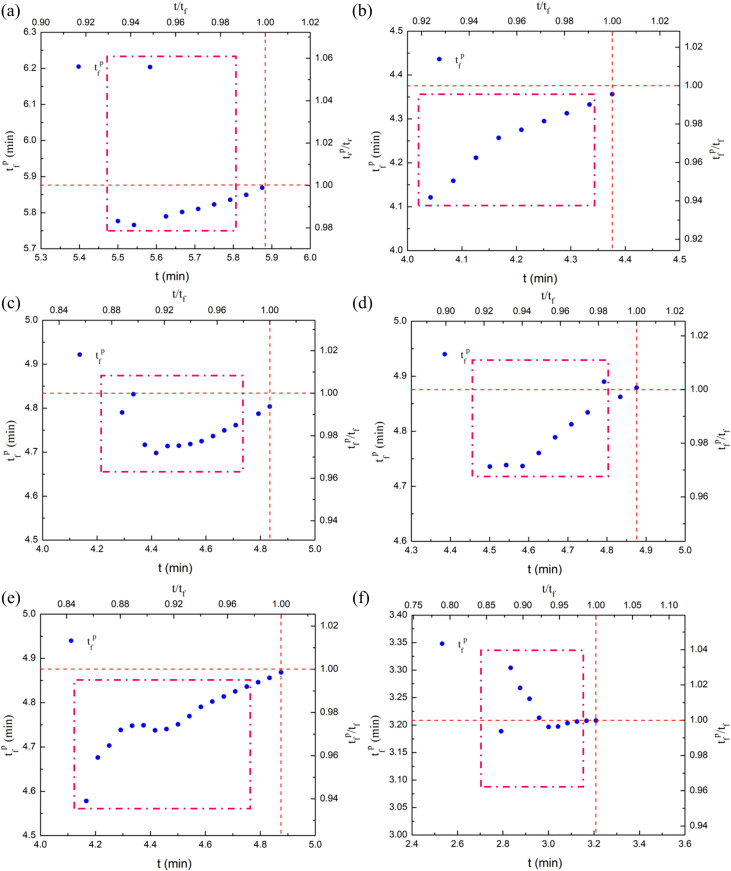
Failure time prediction results of thawed sandstone with different freezing temperatures: **(a)** 20°C; **(b)** −10°C; **(c)** −15°C; **(d)** −20°C; **(e)** −25°C; and **(f)** −30°C. *t* is the monitoring time corresponding to data point under uniaxial compression; *t*_*f*_^*p*^ denotes the predicted time of imminent rock failure; *t*_*f*_ represents the actual time of rock failure; *t/t*_*f*_ represent the ratio of monitoring/ actual failure time; *t*_*f*_^*p*^/*t*_*f*_ represent the ratio of predicted/ actual failure time.

The critical acceleration process of the correspond variables before thawed sandstone failure contains the key information for the catastrophic prediction. Based on the critical linear relationship, the data of the proximity destruction tests are selected for the catastrophic failure time prediction by the sliding window (the number of data points in the window remains the same, and the window is gradually slid towards the catastrophic failure point). The rock failure time (*t*_*f*_^*p*^) is calculated according to the calculated power-law singularity index *β*_*1*_ combined with equation (12), and dimensionless time is carried out based on the rock self-destruction time *t*_*f*_. Finally, the prediction results of thawed sandstone instability failure time under different freezing temperatures are obtained, as presented in Fig. 13.

Observing the trend of the data points in the Fif, it can be seen that the prediction results of the experimental data in the early stage of near failure deviate from the intersection point, indicating that the deviation between the predicted value and the actual value is relatively large. After that, as the sliding window continues to move backward (in the later stage of near failure), the prediction results of the experimental data gradually approach to the intersection point, indicating that the prediction results gradually converge to the true value. This illustrates that the experimental data close to the failure point present the critical power-law singular behavior, and the failure prediction exhibits good stability and reliability. By counting multiple samples at different freezing temperatures, the predicted failure time *t*_*f*_^*p*^ obtained by the constructed catastrophe prediction method is very close to the real damage time *t*_*f*_ under the condition of unknown critical power-law singularity index. More specifically, the actual failure time at different freezing temperatures (0, −10, −15, −20, −25, and −30°C) were 5.875 min, 4.376 min, 4.834 min, 4.875 min, and 4.875 min, 4.875 min and 3.209 min, respectively, and the corresponding predicted failure time were 5.869 min, 4.356 min, 4.804 min, 4.879 min, 4.868 min and 3.208 min, respectively. The absolute values of the difference between the predicted value and the actual value were 0.006 min, 0.02 min, 0.03 min, 0.004 min, 0.007 min and 0.001 min, respectively, and the maximum difference (i.e., absolute error) is 1.8s. This may be due to the influence of data monitoring accuracy and data processing error [74], but the relative error is extremely small and within the controllable range. The prediction results are basically consistent with the experimental results. In addition, because the sliding window always selects the latest observation data, which close to the catastrophic failure, the influence of the early deviation from the power-law precursory behavior data can be excluded and gradually approach experimental data which exhibit power-law growth, thereby realizing the real-time prediction of the catastrophic failure time. Therefore, it is feasible to construct a method for predicting the failure time of sandstone in Luohe Formation suffering one freeze-thaw cycle by using the critical power-law singularity behavior. The experimental data are used to verify the proposed prediction method, and the predicted value matches well the actual value, indicating that the prediction results are good and suitable for practical engineering applications.

## 6 Conclusion

In this paper, the uniaxial compression tests were carried out to study the mechanical properties, failure mode and energy evolution of Luohe Formation sandstone which were subjected to one freeze-thaw cycle with different freezing temperatures. A catastrophic failure time prediction mode for the thawed sandstone in Luohe Formation was proposed according to the critical power-law singularity characteristics of response quantity, and the rationality and effectiveness of this prediction model were verified. Based on the obtained results, the major research conclusions are summarized as follows:

(1) The peak strength and elastic modulus of thawed sandstone decrease with the decrease of freezing temperature. Three major failure modes, including axial splitting failure, shear failure and tension-shear mixed failure occurred at different freezing temperatures, and the degree of local rock block peeling and surface particle shedding of sandstone tend to be more significant with decreasing the freezing temperature.
(2) The severity of rock failure is closely related to the strain energy release rate and the dissipated energy increase rate. The strain energy release rate and dissipation energy increase rate tend to decrease with decreasing the freezing temperature, which corresponds to the intensity of rock failure and its failure mode.
(3) With the decrease of freezing temperature, the energy storage limit and total energy at peak point exhibited fluctuations and presented a reduction trend, and dissipated energy also showed gradually decreasing trend, but the variation extent was relatively small.
(4) The energy damage evolution process of the Luohe Formation sandstone after thawing is divided into stages of minor damage, deceleration damage, damage steadily increase, damage accelerated increase, and damage sharply increase based on the evolution characteristics of the energy damage variable rate.
(5) The energy damage variable rate presents a clear critical power-law singularity behavior before the thawed sandstone catastrophic failure, and the critical singularity characteristic value β was about 0.7±0.1. A catastrophic failure time prediction mode for the thawed sandstone in Luohe Formation was presented. and the destruction prediction time (*t*_*f*_^*p*^) is very close to the actual destruction time (*t*_*f*_).

In this paper, indoor freeze-thaw experiments and uniaxial compression test were performed to study energy evolution and catastrophic failure prediction of sandstone in Luohe Formation, which is different from actual practical projects. Field observations and in situ tests must be further studied to forecast catastrophic failure time in actual engineering.
